# Does Kisspeptin Signaling have a Role in the Testes?

**DOI:** 10.3389/fendo.2013.00198

**Published:** 2013-12-30

**Authors:** Hua Mei, Joanne Doran, Victoria Kyle, Shel-Hwa Yeo, William H. Colledge

**Affiliations:** ^1^Jules Stein Eye Institute, University of California, Los Angeles, CA, USA; ^2^Takeda Cambridge Ltd., Cambridge, UK; ^3^Department of Physiology, Development and Neuroscience, University of Cambridge, Cambridge, UK

**Keywords:** kisspeptins, *Gpr54/Kiss1r*, testes, Leydig cells, testosterone secretion, spermatids

## Abstract

Kisspeptins are a family of overlapping neuropeptides encoded by the *Kiss1* gene that regulate the mammalian reproductive axis by a central action in the hypothalamus to stimulate GnRH release. Kisspeptins and their receptor (GPR54 also called KISS1R) are also expressed in the testes but a functional role in this tissue has not been confirmed. We examined which cell types in the testes expressed kisspeptin and its receptor by staining for β-galactosidase activity using tissue from transgenic mice with *LacZ* targeted to either the *Kiss1* or the *Gpr54* genes. Expression of both genes appeared to be restricted to haploid spermatids and this was confirmed by a temporal expression analysis, which showed expression appearing with the first wave of haploid spermatid cells at puberty. We could not detect any kisspeptin protein in spermatids however, suggesting that the *Kiss1* mRNA may be translationally repressed. We tested whether kisspeptin could act on Leydig cells by examining the effects of kisspeptin on the immortalized Leydig cell line MA-10. Although MA-10 cells were shown to express *Gpr54* by RT-PCR, they did not respond to kisspeptin stimulation. We also tested whether kisspeptin could stimulate testosterone release by a direct action on the testes using explants of seminiferous tubules. The explants did not show any response to kisspeptin. The functional integrity of the MA-10 cells and the seminiferous tubule explants was confirmed by showing appropriate responses to the LH analog, human chorionic gonadotropin. These data suggest that kisspeptin signaling does not have a significant role in testes function in the mouse.

## Introduction

Kisspeptins, encoded by the *Kiss1* gene, are an overlapping family of neuropeptides required for activation and maintenance of the mammalian reproductive axis [for review, see Ref. (1)]. Kisspeptins are encoded as a 145-amino-acid precursor protein in humans that is cleaved into shorter peptides (Kp54, Kp14, Kp13, and Kp10) that share a common RF-amide C-terminal decapeptide sequence. They all act as potent stimulators of GnRH release by signaling through the G-protein coupled receptor, GPR54 (also called KISS1R) expressed by GnRH neurons. Disruption of kisspeptin signaling causes hypogonadotropic hypogonadism in mice and humans (2–7). Mutant mice do not undergo sexual maturation at puberty and have low gonadotropic and sex steroid hormones levels caused by defective GnRH secretion from the hypothalamus. Conversely, activating mutations of *GPR54* cause precocious puberty in humans (8).

In addition to the role of kisspeptins in the central regulation of the reproductive axis, *Gpr54* expression has been detected in the testes of humans (9, 10), mice (3), rats (11), and frogs (12) raising the possibility that kisspeptins may also act at this location. Kisspeptins have been immunolocalized to Leydig cells in mice (13) and kisspeptin and GPR54 have been detected in human sperm, mainly localized to the head, neck, and the flagellum midpiece (14).

Although the expression profile of *Kiss1* and *Gpr54* suggests that kisspeptin signaling might have a role in the testes, very little has been done to test this hypothesis. The data to support a role for kisspeptin in the testes is largely circumstantial and based on discrepancies between the normally direct relationship of LH and testosterone levels. For example, in rats, chronic (13 days) subcutaneous administration of kisspeptin reduced testosterone secretion without a significant decrease in plasma LH (15). In Rhesus monkeys, continuous intravenous infusion of human kisspeptin over 4 days maintained plasma testosterone levels even after the LH stimulation levels had fallen (16). When circulating testosterone levels were expressed relative to LH levels, the [T]:[LH] ratios were significantly higher in the morning in the high dose kisspeptin treatment group compared to the vehicle group. This led to the suggestion that kisspeptins might augment the LH-induced secretion of testosterone. Support for this has come from kisspeptin administration in Rhesus monkeys pre-treated with acyline, a GnRH receptor antagonist, to allow the intratesticular actions of kisspeptin to be evaluated without the confounding effects of LH release from the pituitary (17). Kisspeptin administration significantly increased human chorionic gonadotropin (hCG)-stimulated testosterone levels in acycline treated monkeys compared to hCG treatment alone (17) suggesting that kisspeptin might enhance LH responses in Leydig cells.

To further investigate the possible function(s) of kisspeptin in the mouse testes, we used transgenic mice with *Kiss1* and *Gpr54* alleles targeted with a *LacZ* reporter gene to define the testicular cell expression profile of these genes. We also tested whether kisspeptins can stimulate testosterone release from an immortalized mouse Leydig cell line and from primary testes explants in culture.

## Materials and Methods

### Mouse lines and maintenance

The 129S6/SvEv mutant mice with a targeted disruption of the *Gpr54* or *Kiss1* genes were generated as described previously (2, 5). All mice were maintained on a 12:12-h light-dark cycle (light on between 6:30 a.m. and 6:30 p.m.) with *ad libitum* access to food and water. Experimental procedures were performed under authority of a Home Office Project License and approved by a Local Ethics Committee.

### MA-10 cell culture

The mouse Leydig tumor cell line MA-10 (18) was a generous gift from Dr. Mario Ascoli (University of Iowa, Iowa City, IA, USA). The MA-10 cells were maintained in RPMI-1640 medium (Sigma-Aldrich, Dorset, UK) containing 10% horse serum (Sigma-Aldrich, Dorset, UK) and 10% newborn calf serum (Sigma-Aldrich, Dorset, UK), and the cells were grown at 37°C in an humidified atmosphere of 5% CO_2_. The growth medium was refreshed every 2 days to provide sufficient nutrition for cell growth.

### RT-PCR gene expression analysis of MA-10 cells

Total RNA was isolated from MA-10 cells using a NucleoSpin^®^ RNA II kit (Cat No: 740955, MACHEREY-NAGEL GmbH & Co. KG) following the manufacturer’s protocol. The time of the on-column DNA digestion was extended from 15 to 45 min to ensure complete removal of genomic DNA. The RNA was reverse transcribed into cDNA using SuperScript III Reverse Transcriptase (Cat No: 18080-044, Invitrogen, UK) following the protocol provided by the manufacturer. Standard PCR was performed as follows: the samples were denatured for 5 min at 95°C and amplified for 44 cycles (30 s at 93°C, 1 min at 60°C, and 2 min at 70°C). The primer sequences were: *Kiss1* (Forward: tgctgcttctcctctgtgtcg; Reverse: gccgaaggagttccagttgta, 310 bp product), *Gpr54* (Forward: gccttcgcgctctacaacctgctg; Reverse: aaggcatagagcagcggattgagc, 367 bp product), *GnRH* (Forward: cggcattctactgctgactgt; Reverse: catcttcttctgcctggcttc, 229 bp product), β-actin (Forward: ctgtattcccctccatcgtg; Reverse: gggtcaggatacctctcttgc, 113 bp product). RNA without a reverse transcription step was used as a negative control for identification of genomic DNA contamination and cDNA from wild-type hypothalamus was used as a positive control for *Kiss1* amplification.

### X-gal staining of testes sections

Testes were fixed in 1% paraformaldehyde/PBS overnight at 4°C, cryoprotected with 30% sucrose/PBS overnight at 4°C, and cryosectioned at 20 μm onto poly-lysine coated slides. Sections were air dried at room temperature, rehydrated in PBS and β-galactosidase activity detected using a *LacZ* staining solution [1 mM MgCl_2_, 1 mg/ml X-gal (5-bromo-4-chloro-3-indolyl β-d-galactopyranoside), 5 mM potassium ferricyanide, 5 mM potassium ferrocyanide in PBS] at 37°C overnight, and counterstained with 1% Saffronin.

### Immunohistochemistry to detect kisspeptin expression

Testes were fixed in 4% paraformaldehyde/Tris-buffered saline (TBS) for 5 h at room temperature and transferred to 30% sucrose/TBS overnight at 4°C. The testes were then cryosectioned at 15 μM, air dried at room temperature, rehydrated in TBS, and slide-mounted immunohistochemistry was performed to detect kisspeptin expression. Polyclonal antibody AC566 raised in rabbits against mouse Kp10 was a generous gift from Alain Caraty, Tours, France. Characterization and specificity of AC566 has been described previously (5, 19–21).

Sections were treated with 3% hydrogen peroxide for 15 min to quench endogenous peroxidase and then washed in TBS. To visualize kisspeptin expression, sections were incubated with the antibody at 1:2000 dilution for 8 h at room temperature. For secondary antibody labeling, sections were incubated with biotinylated goat anti-rabbit (1:100; Cat No: BA-1000, Vector Laboratories, Peterborough, UK) immunoglobulins at room temperature followed by incubation with Vector avidin-peroxidase (1:50; Cat No: PK-4000, Vector Laboratories, Peterborough, UK). Finally, the sections were rinsed and immunoreactivity was revealed with glucose-oxidase and nickel-enhanced diaminobenzidine hydrochloride (12.5 mg/ml). Sections were counterstain with hematoxylin, dehydrated in ethanol followed by Histoclear, and then coverslipped with DPX.

### Progesterone release experiments from MA-10 cells

The MA-10 cells were seeded at 2.5 × 10^5^ cells/well (24-well plates) 24 h before the hormone treatment. The cells were treated with increasing concentrations of Kp10 (human Metastin 45–54) (1, 10, or 20 μM) (Cat No: M2816, Sigma-Aldrich, Dorset, UK) or Kp10 followed by hCG (0.012 IU/ml as the final concentration) (Cat No: CG5, Sigma, Saint Louis, MO, USA). PBS was added as a negative control. Each condition was tested in triplicate. After 4 h, the media was collected for progesterone measurement. After collection of media, the MA-10 cells were rinsed twice with PBS and lysed in 1× lysis buffer (reporter lysis buffer, Cat No: E397A, Promega, UK) by a freeze-thaw cycle. The lysate was briefly centrifuged and the protein content of the supernatant determined with a Bio-Rad Bradford Assay following the standard protocol.

### Primary culture of testes explants

The testes from adult wild-type mice were cut into two pieces (approximately 40 mg/piece) without removing the tunica and each piece was cut and flattened to a 1-mm thickness on a Nylon membrane (Cat No: 1417240, Boehringer-Mannheim, Indianapolis, IN, USA) floating in phenol red-free DMEM medium (Cat No: 21063, GIBCO) (300 μl/well for 12-well plate) supplemented with 1× penicillin/streptomycin. The tissues were immediately treated with vehicle, Kp10 (1 μM), hCG (0.6 IU/ml), or a mixture of Kp10 (1 μM) and hCG (0.6 IU/ml), respectively. Each condition was tested in at least four repeat wells. The tissues were cultured at 37°C in an atmosphere of 5% CO_2_ and the media collected at different time points. Fresh media was added at each time point after media collection.

### Hormone assays

Testosterone and progesterone were measured using ELISA kits (Cat No: EIA1559 and EIA1561 from DRG International, USA) according to the manufacturer’s instructions. The testosterone ELISA kit had a sensitivity of 0.083 ng/ml, an inter-assay variation of 6.7%, and an intra-assay variation of 3.3%. The progesterone ELISA kit had a sensitivity of 0.045 ng/ml, intra-assay variation of 7%, and inter-assay variation of 5%.

## Results

### *Gpr54* and *Kiss1* are expressed in the mouse testes

**Figure 1:**
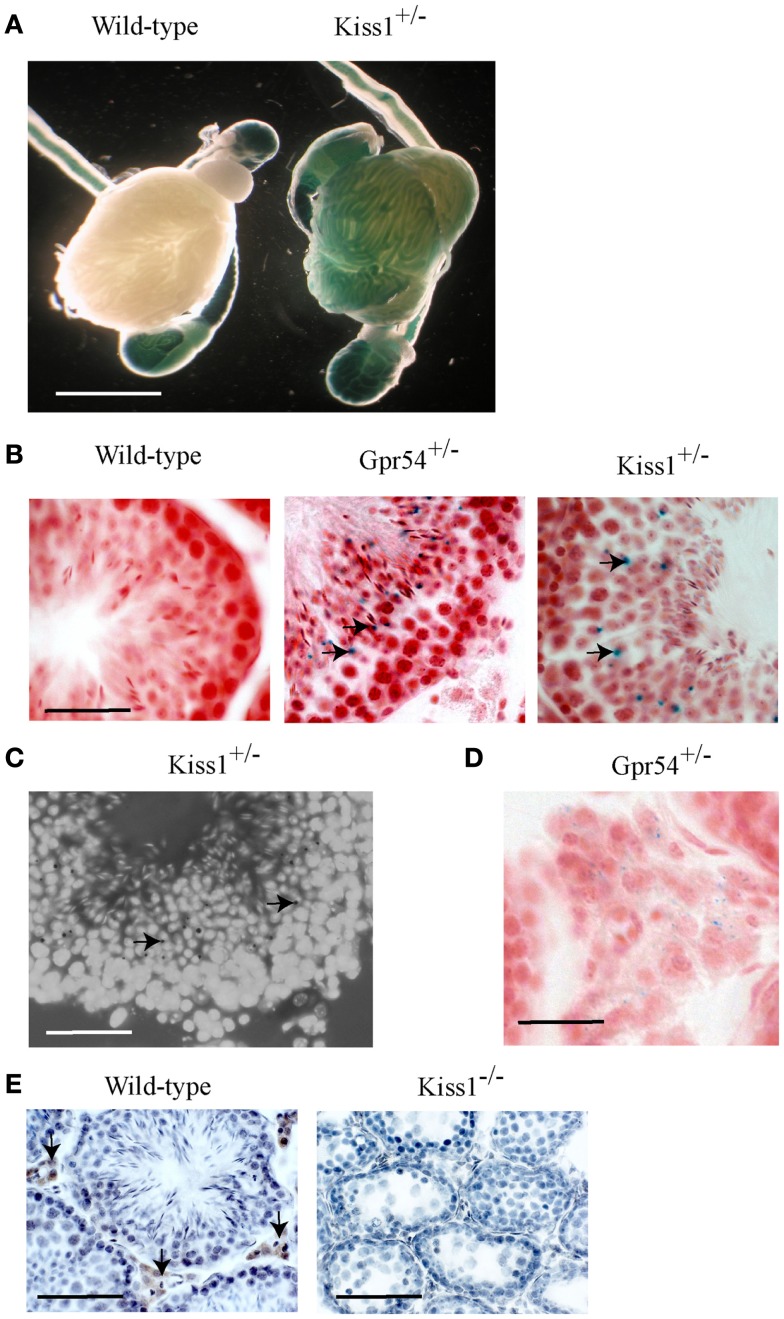
***Kiss1* and *Gpr54* expression in the mouse testes**. **(A)** Expression of *Kiss1* in seminiferous tubules of the testes visualized by X-gal staining (blue) for β-galactosidase activity. Note the non-specific staining in the epididymis and vas deferens of the wild-type testes. Scale bar = 5 mm. **(B)** Cryosections of testes from adult wild-type, *Gpr54*^+/−^, and *Kiss1*^+/−^ mice showing expression (arrowed) localized to spermatid cells of seminiferous tubules. Sections were stained for β-galactosidase activity (blue) and counterstained with Saffronin (red). Scale bar = 100 μm. **(C)** Testes cryosection from *Kiss1*^+/−^ mice stained for β-galactosidase activity (black dots, arrowed) and counterstained with DAPI to visualize cell nuclei illustrating clearer expression in spermatid cells. Scale bar = 100 μm. **(D)** Low expression of *Gpr54* in Leydig cells visualized by X-gal staining. Scale bar = 50 μm. **(E)** Kisspeptin immunoreactivity localized to Leydig cell in wild-type mice (arrowed) but not *Kiss1* mutant mice. Scale bar = 200 μm.

The *Gpr54* and *Kiss1* alleles in the transgenic mice have been tagged with a *LacZ* gene that allows their gene expression patterns to be visualized by staining for β-galactosidase activity. Staining was observed within seminiferous tubules from both *Kiss1*^+/−^ (Figure 1A) and *Gpr54*^+/−^ mice (data not shown) but not in wild-type mice (Figure 1A). Background staining was observed in the epididymis and the vas deferens of wild-type mice as the epithelial cells in these tissues express an endogenous galactosidase-like enzymatic activity. To define the cells in which *Kiss1* and *Gpr54* are expressed, cryosection of testes were stained for β-galactosidase activity. The staining in cryosections was localized to the region of the seminiferous tubules that contained round spermatids (arrowed in Figures 1B,C). The spermatids are easily recognized as they have smaller nuclei than spermatocytes and are four-times more abundant as they have just completed meiosis. Very faint β-galactosidase staining was also found in the Leydig cells in the *Gpr54*^+/−^ mice (Figure 1D) but not in the *Kiss1*^+/−^ mice (data not shown).

**Figure 2:**
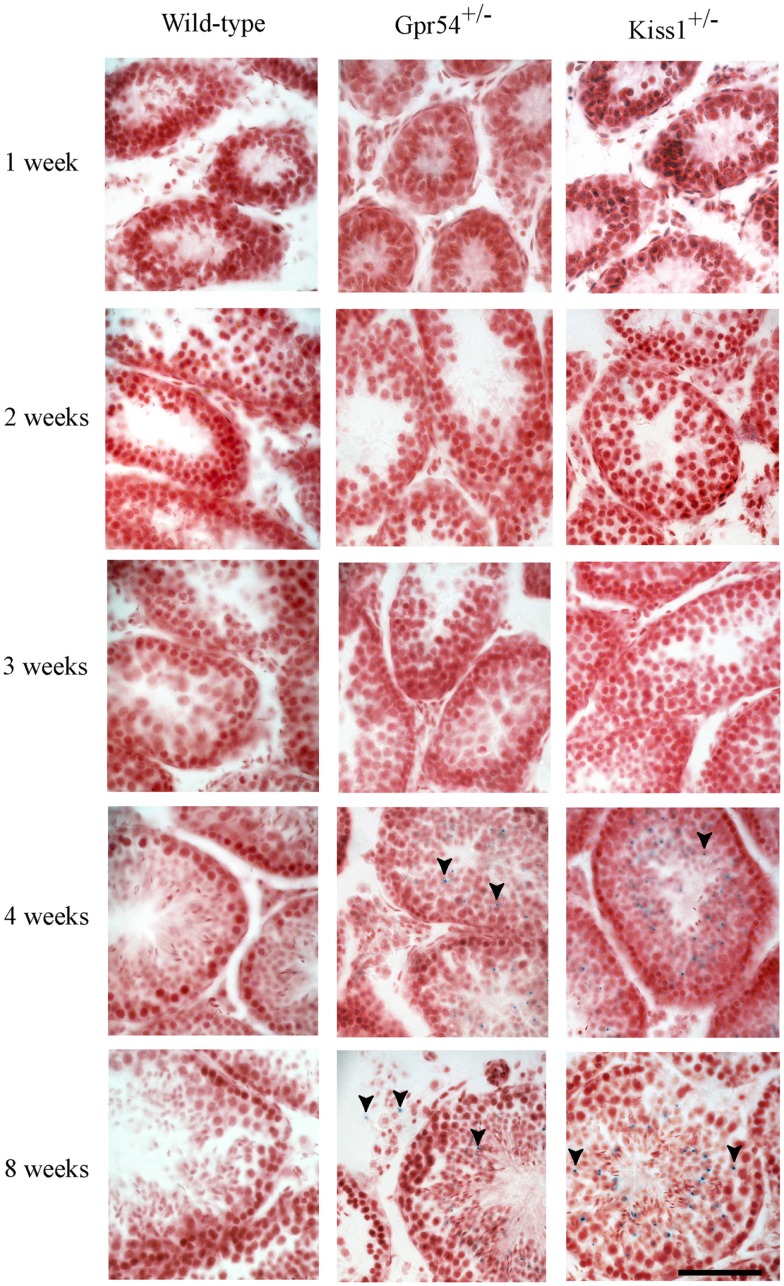
**Developmental time course of *Gpr54* and *Kiss1* expression in the mouse testes**. Testes of wild-type (WT), *Kiss1*^+/−^ and *Gpr54*^+/−^ mice at different ages were cryosectioned, stained for β-galactosidase activity, and counterstained with Saffronin. Blue dots (arrowed) indicate expression of the *Kiss1* and *Gpr54* genes. All photographs are at the same magnification. Scale bar = 50 μm.

To confirm that the β-galactosidase expression was localized to spermatid cells, the time point at which expression was first observed during the first spermatogenic cycle was determined. Expression of *Kiss1* and *Gpr54* could not be observed prior to 3 weeks of age but staining was seen from 1 month of age which corresponds to the time at which the spermatids first appear in mice (Figure 2).

Kisspeptin protein expression in the mouse testes was visualized using a well characterized rabbit antiserum highly specific for mouse Kp10 (20). Strong immunoreactivity was found in Leydig cells with no staining in spermatids (Figure 1E). As a control for antibody specificity, testes sections from *Kiss1* mutant mice lacking kisspeptin protein were used and no immunoreactivity was observed (Figure 1E). This Leydig staining may be non-specific however, as no kisspeptin protein was detected in *Gpr54* mutant mice (data not shown).

### Kp10 does not stimulate steroidogenesis in the leydig cell line, MA-10

To test whether Kp10 could stimulate testosterone release, the mouse Leydig cell line, MA-10, was used (18, 22). MA-10 cells, like normal Leydig cells, express LH receptors and respond to hCG stimulation. MA-10 cells have low expression and activity of P450c17 that is the enzyme that converts progesterone into 17-OH progesterone and finally into testosterone, thus MA-10 cells produce progesterone as the principle steroid hormone instead of testosterone (18, 22).

**Figure 3:**
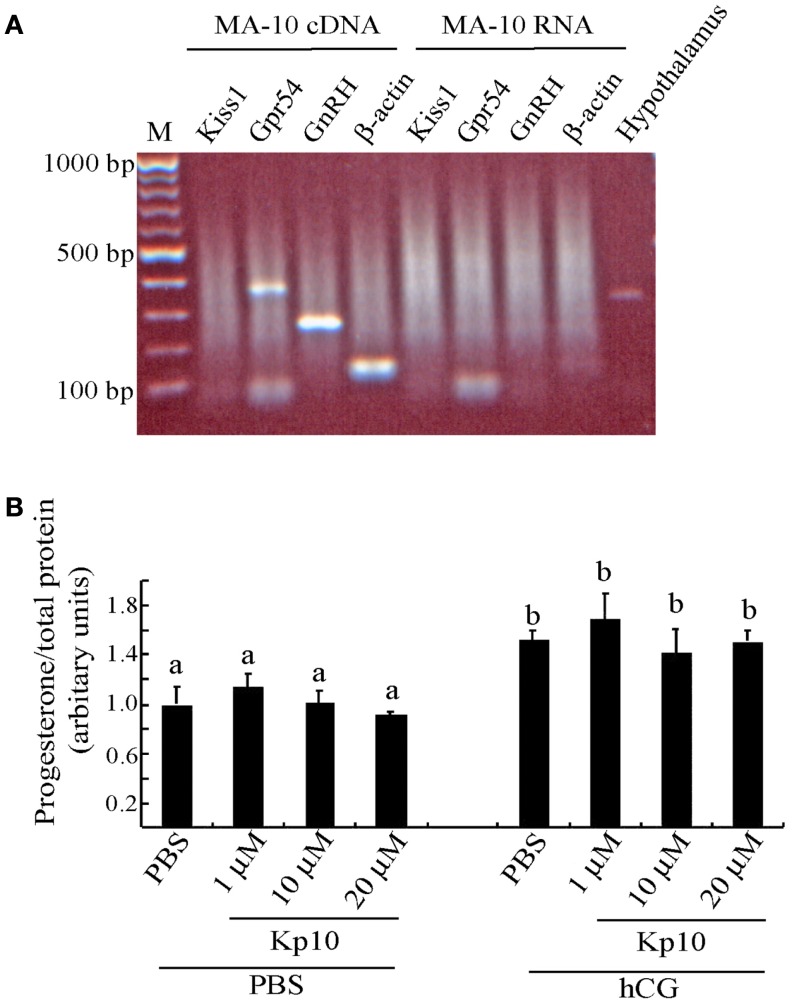
**The MA-10 Leydig cell line expresses *Gpr54* but do not respond to Kp10 treatment**. **(A)** RT-PCR gene expression analysis in the MA-10 Leydig cell line. Expression of *Gpr54, GnRH*, and *β-actin* was detected in cDNA from the MA-10 cells but *Kiss1* expression was not detected. Hypothalamic cDNA was included as a positive control for *Kiss1* expression. **(B)** Effect of Kp10 on stimulating progesterone release from the MA-10 cell line. The MA-10 cells were stimulated with the hormones indicated and media collected after 4 h and tested for progesterone. Different letters (a and b) indicate statistically significant differences between groups (*P* < 0.05, one way ANOVA with a Tukey comparison post-test).

The MA-10 cells were examined for expression of *Kiss1* and *Gpr54* to determine whether they might be capable of responding to kisspeptins. There was a PCR product for *Gpr54* (Figure 3A), indicating that MA-10 cells endogenously express this gene but there was no detectable *Kiss1* expression in the MA-10 cells. The MA-10 cell also expressed GnRH and β-actin transcripts (Figure 3A). No products were observed when non-transcribed RNA was used as the template, indicating that the RNA was free of genomic DNA contamination. A *Kiss1* product was observed when hypothalamic cDNA was used from wild-type mice as a positive control.

As the MA-10 cells expressed the kisspeptin receptor, they were tested to see whether Kp10 could stimulate progesterone release. The cells were divided into two experimental groups. The first group was treated with increasing concentrations of human Kp10 followed by PBS, and the second group was treated with Kp10 followed by hCG to examine possible synergistic effects. After 4 h, the media was assayed for progesterone, which was normalized to the protein content of the cell lysate to correct for variations in cell number. No significant difference in progesterone release was found between the vehicle (PBS) treatment and any of the three concentrations of Kp10 (Figure 3B), indicating that Kp10 cannot enhance progesterone release from the Leydig cell line even at a high concentration (20 μM). There was also no significant difference in progesterone release between the cells treated with hCG alone or those treated with hCG and Kp10 (Figure 3B), which suggests that Kp10 has no synergistic effect on progesterone release from MA-10 cells activated by hCG. However, there was a significant difference (*P* < 0.05) in progesterone release between the cells treated with PBS and those treated with hCG (Figure 3B), which indicates the functional responsiveness of the cells to hormonal stimulation.

### Kp10 does not stimulate testosterone release from testes tissue culture explants

**Figure 4:**
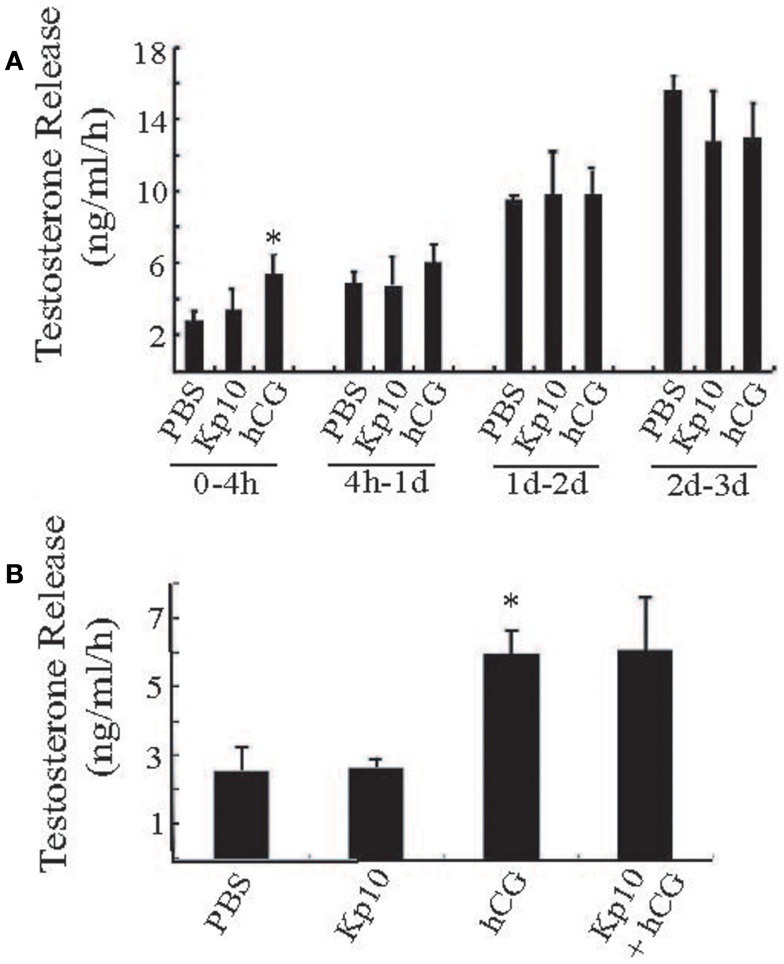
**Testing Kp10 stimulation of testosterone release from fresh testes explants**. **(A)** Time course of testosterone release from explanted mouse testes tissue. Pieces of adult mouse testes were cultured in PBS, Kp10 (1 μM), or hCG (0.6 IU/ml), respectively. The media was collected at the time points indicated, and testosterone levels measured by an ELISA. **(B)** Evaluation of synergistic action of Kp10 on hCG-mediated testosterone release. Pieces of adult mouse testes were cultured in PBS, Kp10 (1 μM), hCG (0.6 IU/ml), or a mixture of Kp10 (1 μM) and hCG (0.6 IU/ml). The media was collected after 4 h incubation and testosterone levels measured by an ELISA. *n* ≥ 4 for all columns. *Statistically significant difference (*P* < 0.05) between the hCG and the vehicle control group (PBS) (unpaired *t*-test with Welch’s correction).

To examine the possible action of Kp10 in a more physiological system, we tested whether Kp10 could stimulate testosterone release from primary explants of mouse testes. Pieces of adult wild-type mouse testes of similar weight (approximately 40 mg/piece) were treated with vehicle (PBS), Kp10 (1 μM), hCG (0.6 IU/ml), or a mixture of Kp10/hCG. Each condition was repeated with at least four samples. The media was collected at different time intervals for testosterone measurements. During the 0- to 4-h time period, the testosterone released after hCG treatment was significantly higher than that with the vehicle (PBS) treatment (Figure 4A), indicating that the cultured testes maintained the ability to respond to hormone stimulation. However, there was no obvious stimulation of testosterone release after Kp10 treatment. Also, the testosterone released in the PBS and hCG groups was not significantly different at incubation times >4 h due to increased unstimulated testosterone release (Figure 4A). Therefore, a 4-h incubation time was used to test whether there was any synergy between Kp10 and hCG in stimulating testosterone release (Figure 4B). Once again, testosterone release after hCG treatment was significantly higher than after PBS treatment (Figure 4B). No difference in testosterone release was detected between the testes fragments cultured in PBS or Kp10. There was also no difference in testosterone release between testes treated with hCG only and testes treated with hCG and Kp10 together. These data indicate that Kp10 has no effect on testosterone release from adult mouse testes and it has no synergistic action on testosterone release stimulated by hCG.

## Discussion

Kisspeptin neuropeptides are important central regulators of the mammalian reproductive axis with kisspeptin neurons acting upstream of GnRH neurons to stimulate GnRH release. In addition to this central role however, the expression profiles of *Kiss1* and *Gpr54* suggest that they may also have a function in peripheral tissues including the testes. We have shown using expression of a gene targeted *LacZ* reporter gene, that *Kiss1* and *Gpr54* are expressed by round spermatid cells in the mouse testes. Expression profiling during postnatal gonadal maturation confirmed this as the expression only started to emerge after 1 month of age, which is the time when the spermatids first appear. As both *Kiss1* and *Gpr54* were found to be expressed in spermatids, this raises the possibility that autocrine or paracrine kisspeptin signaling might be involved in spermiogenesis.

Round spermatid cells have just completed meiosis and will subsequently undergo the structural changes required to produce spermatozoa. During this structural remodeling, most of the cytoplasm is removed from the spermatids by the Sertoli cells, which will result in loss of β-galactosidase activity, which might explain why we do not observe staining in elongating spermatids and spermatozoa. Similarly, this cytoplasmic removal would remove any kisspeptin protein but GPR54 should be retained by virtue of its location in the plasma membrane. Indeed, GPR54 has been detected in the head region of human sperm and addition of kisspeptin can produce a modest rise in [Ca^2+^]_i_ and sperm motility (14).

The functional significance of *Kiss1* and *Gpr54* expression in spermatids and sperm is still not clear however. The infertility of the *Kiss1* and *Gpr54* mutant mice prevents performing functional tests with mutant sperm. It might be possible to initiate spermatogenesis in the mutant mice with pulsatile FSH and subcutaneous testosterone delivery. Although we have shown that *Kiss1* and *Gpr54* mutant mice can initiate a low level of spermatogenesis when given a chow diet containing phytoestrogens (23), the number of sperm that can be isolated from the vas deferens and epididymis is too small for functional studies. It is noteworthy, however, that several male patients with mutations in *GPR54* and hypogonadotropic hypogonadism have responded to exogenous hormone treatment and achieved fertility [for review, see Ref. (24)] suggesting that in humans, GPR54 function is not essential for sperm function.

There is an important caveat to this expression data however. Although the *LacZ* expression indicates that the *Kiss1* promoter is transcriptionally active in round spermatid cells, we could not detect kisspeptin immunoreactivity using a validated antibody capable of visualizing kisspeptin in the hypothalamus of mice (25). It is possible that the expression level of the kisspeptin protein is below the limits of detection and that X-gal staining for β-galactosidase activity is more sensitive. Alternatively, it is possible that *Kiss1* transcripts are not translated into protein in spermatid cells. Several gene transcripts encoding proteins required for late spermiogenesis are expressed in round spermatids and translationally repressed until the elongating spermatid stage (26). Translationally repressed mRNAs have unusually long poly(A) tails of approximately 180 nt and translation is associated with shortening of these tails (27). The presence of long poly(A) tails on *Kiss1* transcripts, which are not subsequently shortened, might provide a mechanism for the proposed translational repression in spermatids.

We detected kisspeptin immunoreactivity in Leydig cells of wild-type mice similar to that reported by Anjum and colleagues (13). The specificity of this immunoreactivity was suggested by absence of staining in *Kiss1* mutant mice, which do not produce any kisspeptin protein (5). This notwithstanding, we believe that the kisspeptin immunoreactivity found in the Leydig cells may not be authentic for the following reasons. Firstly, we did not detect *Kiss1* promoter activity in Leydig cell by β-galactosidase staining in *Kiss1*^+/−^ mice. Secondly, we did not detect *Kiss1* transcripts by RT-PCR in the immortalized Leydig cell line MA-10 although this may be a consequence of the cell immortalization process and the tendency for *Kiss1* expression to be suppressed during cell transformation and tumorigenesis. Finally, we failed to detect kisspeptin immunoreactivity in the Leydig cells of *Gpr54* mutant mice, which can produce kisspeptin protein. We believe that the staining pattern observed in the Leydig cells of the wild-type mice is an artifact perhaps associated the high levels of steroidogenesis in these cells, which does not occur in *Kiss1* or *Gpr54* mutant mice.

We also observed a very low level of β-galactosidase staining in Leydig cells from *Gpr54*^+/−^ mice suggesting that these cells might express GPR54 protein. This was consistent with our detection of *Gpr54* transcripts in the immortalized mouse Leydig cell line MA-10. Unfortunately, there are no anti-GPR54 antibodies with sufficient specificity to confirm expression of the endogenous GPR54 protein in the Leydig cells.

If there was co-expression of GPR54 and kisspeptin in Leydig cells, this would allow local autocrine or paracrine action within the testes. Previously published work has suggested that kisspeptins are able to enhance testosterone release after LH stimulation (17). We therefore examined whether Kp10 was able to stimulate testosterone release from the MA-10 cell line as well as testes fragments in culture. We found no evidence that Kp10 could directly stimulate testosterone release or enhance the actions of LH. This is in contrast to the recent data that kisspeptin administration significantly increased hCG-stimulated testosterone levels in acyline treated Rhesus monkeys compared to the responses with hCG treatment alone (17). As the acyline inhibits endogenous LH secretion from the pituitary, these responses suggest a direct, synergistic action of kisspeptin on the testes. The reason for the difference from our data is not known, but apart from a species difference, it might be that the enhancement by kisspeptin requires a sub-threshold level of LH stimulation and the concentration of hCG that we used was too high. It would be informative to test Kp10 responses to a lower range of hCG treatments in the testes explants. It is noteworthy, however, that Huma and colleagues have found that an intravenous injection of the kisspeptin antagonist p234 does not alter plasma testosterone levels in adult Rhesus macaques (28) suggesting that any action of kisspeptin on the testes is small. This conclusion is consistent with the observation that fertility can be restored in *Gpr54* mutant mice by expression of a *Gpr54* transgene in GnRH neurons (29) indicating that GPR54 expression in the testes is also not essential for fertility in mice.

In summary, we have shown that the *Kiss1* and *Gpr54* are both expressed in round spermatid cells of the mouse testes and *Gpr54* is expressed by Leydig cells but we have not found any supporting data that kisspeptin signaling in the testes has a major role in spermatogenesis or testosterone secretion in the mouse.

## Conflict of Interest Statement

The authors declare that the research was conducted in the absence of any commercial or financial relationships that could be construed as a potential conflict of interest.
